# First complete genome of a fowlpox virus isolated in Bangladesh reveals Asian lineage and clade A1

**DOI:** 10.1128/spectrum.03114-25

**Published:** 2026-05-29

**Authors:** Anandha Mozumder, Roni Mia, Anupam Das, S. M. Nazmul Hasan, Jebin Tasmin, Raduyan Farazi, Md. Salauddin, M. Shaminur Rahman, Sharmin Akter, Sukumar Saha, Tofazzal Islam, Md. Golzar Hossain

**Affiliations:** 1 Department of Microbiology and Hygiene, Bangladesh Agricultural University 54492, Mymensingh, Bangladesh; 2 Department of Microbiology and Public Health, Khulna Agricultural University 683038, Khulna, Bangladesh; 3 Department of Microbiology, Jashore University of Science and Technology 421984, Jashore, Bangladesh; 4 Department of Physiology, Bangladesh Agricultural University 54492, Mymensingh, Bangladesh; 5 Institute of Biotechnology and Genetic Engineering, Gazipur Agricultural University 198780, Gazipur, Bangladesh; Oklahoma State University College of Veterinary Medicine, Stillwater, Oklahoma, USA

**Keywords:** fowlpox virus, Bangladesh, genome sequencing, phylogenetic analysis, mutations

## Abstract

Fowlpox virus (FPV) is an economically important avian pathogen that continues to cause recurrent outbreaks in poultry worldwide, including Bangladesh, despite routine vaccination. This study aimed to isolate, sequence, and characterize the complete genome of a field FPV strain from Bangladesh. This study aimed to isolate, sequence, and characterize the complete genome of a field FPV strain from Bangladesh to provide insights into its evolutionary characteristics and genomic diversity, thereby improving the understanding of FPV evolution in South Asia. Samples from layer flocks were examined by PCR, and the virus was isolated in embryonated chicken eggs (ECEs). The viral genome was sequenced using next-generation sequencing and compared with global strains. Phylogenetic analyses were conducted using complete genomes and the P4b gene, while mutational, structural, and antigenicity analyses of key viral proteins (serpin, V-type Ig domain, protein tyrosine phosphatase, and ankyrin repeat proteins) were performed using bioinformatics tools. The FPV showed characteristic pock lesions in chorioalantoic membrane of ECEs. The isolate, FPV_MGH-AM01, comprised 288,539 bp with 30.9% GC content and showed 99.69%–99.89% nucleotide identity with global strains, confirming high conservation. Phylogenetic analysis clustered it with South Korean strains, reflecting Asian lineage characteristics, while P4b gene analysis confirmed its placement in clade A1. The genome retained regions deleted in some Chinese and French isolates. A total of 71 mutated genes were identified, including 54 with amino acid substitutions. Structural and physicochemical properties of major proteins were largely conserved, although the V-type Ig domain protein displayed altered antigenicity. In conclusion, this first complete genome of FPV from Bangladesh demonstrates high conservation with various mutations. These findings provide a genomic framework for understanding FPV evolution in South Asia and highlight the need for genomic surveillance to guide vaccine design and control strategies.

**IMPORTANCE:**

Fowlpox virus (FPV) remains a persistent threat to the global poultry industry, causing substantial economic losses despite widespread vaccination. This study presents the first complete genome sequence of an FPV isolate from Bangladesh, providing critical insights into the genetic makeup, evolutionary lineage, and protein-level variations of circulating field strains in South Asia. By combining whole-genome sequencing, phylogenetic analysis, mutational profiling, and structural characterization of key immunomodulatory proteins, this work establishes a comprehensive genomic framework for FPV in Bangladesh. The identification of amino acid substitutions in proteins, such as serpin, V-type Ig domain, protein tyrosine phosphatase, and ankyrin repeat proteins, and the assessment of their structural and antigenic implications offer valuable information for understanding viral evolution, host-pathogen interactions, and potential vaccine escape. Overall, these findings enhance our understanding of FPV diversity, facilitate genomic surveillance, and provide a foundation for evidence-based vaccine design and control strategies.

## INTRODUCTION

Fowlpox virus (FPV) infects more than 200 avian species, including both domesticated and wild birds, such as chickens, turkeys, pigeons, canaries, and wild passerine birds, etc. (Table 1) (1, 2). It poses a significant economic threat to the global poultry industry, including Bangladesh, where the disease remains endemic and affects poultry production systems, ranging from commercial farms to backyard flocks (3, 4). FPV can infect chickens of all ages, sexes, and breeds, causing substantial economic losses through reduced egg production, impaired growth, and increased mortality (5).

**TABLE 1:** Representative avian species infected with avipoxvirus and associated clinical manifestations

Host species	Clinical symptoms	Geographic region	References
Chicken	Cutaneous lesions, diphtheritic lesions	Worldwide (Asia, Africa, Europe)	(6)
Turkey	Cutaneous nodules, respiratory lesions	Europe, North America	(7)
Pigeon	Nodular lesions on eyelids and beak	Asia, Middle East	(8)
Canary	Cutaneous lesions	Europe	(9)
Wild passerine bird	Skin lesions	Global	(10)

Clinically, fowlpox manifests in two forms. The cutaneous form is characterized by discrete, wart-like proliferative lesions on the skin particularly in the comb, wattles, eyelids, beak, and legs, while the diphtheritic form presents as moist, necrotic lesions on the mucous membranes of the oral cavity and upper respiratory tract. The diphtheritic form is particularly severe and may lead to death by asphyxiation (11). In severe cases, infected birds may exhibit reduced feed intake, decreased egg production, weight loss, and increased mortality. Mixed infections with both forms can also occur and are often associated with more severe disease outcomes (6). Transmission occurs primarily through horizontal spread via aerosols, poultry house dust, dried scabs, and feather debris, while insects may occasionally contribute to environmental dissemination (3). FPV is highly resistant to desiccation and can persist for extended periods on perches and in dried scabs, facilitating long-term survival and recurrent outbreaks (12). Importantly, FPV infection can be prevented through the use of live-attenuated vaccines (5, 13).

FPV belongs to the family *Poxviridae*, genus *Avipoxvirus*, and possesses a large double-stranded DNA genome of approximately 300 kbp (14). The genome contains two inverted terminal repeats flanking the core coding region and encodes around 260 open reading frames (ORFs) (2). Like other avipoxviruses, FPV encodes a wide array of proteins involved in viral replication, immune evasion, and pathogenesis. Many of these proteins counteract host antiviral responses, modulate signaling pathways, and ensure efficient viral persistence (15). Previous studies have shown that poxviruses produce diverse immunomodulatory proteins-including serpins, ankyrin repeat proteins, immunoglobulin-like domain proteins, and tyrosine phosphatases-that enhance virus–host interactions and virulence (2, 16, 17). A highly conserved 75.2 kDa core protein encoded by the *P4b* gene is typically used for genetic identification and classification of viral strains (18) .

Based on nucleotide sequence divergence and insertion/recombination events, FPV genomes can be classified into different clades (A, B, and C) and subclades (A1, A2, A3, and A4) (1, 14). Both field strains (identified between the 1960s and 2019) and vaccine strains have shown considerable genetic diversity across different regions (14). Genomic characterizations of FPV strains in neighboring countries, including India, Nepal, Iraq have also revealed significant genomic variation (13, 19, 20). Although FPV vaccination is routinely practiced, infections continue to be reported in various regions of Bangladesh (5). To date, most studies in Bangladesh have focused on clinical identification, molecular detection, and virus isolation, leaving critical gaps in complete genome sequencing and comprehensive genomic characterization (4).

Therefore, the present study reports, for the first time, the complete genome sequence of an FPV isolate from a field outbreak in Bangladesh. Furthermore, we performed comprehensive genomic analyses, including phylogenetic classification, mutational profiling, and structural characterization of major viral proteins, to provide novel insights into Bangladeshi FPV diversity and evolutionary dynamics.

## MATERIALS AND METHODS

### Sample collection

Seven wart samples were collected from layer chicken flocks suspected of FPV infection. The warts were excised using sterile scalpels, transferred into labeled Eppendorf tubes containing phosphate-buffered saline (PBS) supplemented with antibiotics, and transported under cool conditions to the Laboratory of Virology, Department of Microbiology and Hygiene, Bangladesh Agricultural University.

### Inoculum preparation

Approximately 5 g of wart tissue was minced with sterile scissors and forceps, then homogenized in PBS using a mortar and pestle with sterile sea sand. The homogenates were subjected to three cycles of freezing and thawing, after which a 10% (w/v) suspension was prepared and centrifuged twice at 3,000 rpm for 15 min (21). The supernatant was treated with gentamicin (500 µg/mL) and nystatin (50 µg/mL) and used for DNA extraction and/or inoculation into embryonated chicken eggs.

### DNA extraction and PCR amplification

Viral DNA was extracted from the inoculum using the TIANamp Genomic DNA Kit (TIANGEN, China) according to the manufacturer’s instructions. The DNA was either used immediately for PCR or stored at −20 °C until use. PCR was performed in a 20 µL reaction mixture containing 0.5 µM of each primer (P2FP-F: 5´-CAGCAGGTGCTAAACAACAA-3´ and P2FP-R: 5´-CGGTAGCTTAACGCCGAATA-3´), 10 µL of TaKaRa Taq Version 2.0 plus dye Master Mix, 7 µL of DNA template, and 1 µL of double-distilled water. Thermal cycling conditions were 94°C for 5 min; 35 cycles of 94°C for 45 s, 48°C for 90 s, and 60°C for 2 min, followed by a final extension at 60°C for 10 min (4). PCR products were visualized on 1.5% agarose gels under UV illumination (Bio-Rad ChemiDoc Go Imaging System).

### Virus isolation

Fertile chicken eggs were collected from a commercial layer farm with no history of FPV vaccination and incubated for 10 days (Incubator and Hatcher, MG800H ECO, Italy). The prepared viral inoculum (0.2 mL) was inoculated into the chorioallantoic membrane (CAM) of 10-day-old embryonated eggs using a sterile 1-mL tuberculin syringe with a 1/2-inch needle. Openings in the shell and air sac were sealed with melted wax, and the eggs were incubated horizontally at 37°C for 5–6 days, with the artificial air sac facing upward. Eggs were candled twice daily, and embryos that died within 24 h were discarded as non-specific deaths. After 5–6 days, embryos were chilled at 4°C–8°C for 1–2 h. The eggs were disinfected with tincture of iodine, and the shell over the air sac was carefully opened with sterile forceps. The CAM and allantoic fluid were harvested for examination of pock lesions and further confirmation by PCR.

### Sequencing and genome assembly

Viral DNA from the isolated FPV was extracted using the TIANamp Virus DNA/RNA Kit (Tiangen, China) and subjected to next-generation sequencing. Sequencing libraries were prepared following a modified iNextEra protocol. DNA tagmentation was performed using Illumina bead-linked transposomes (Illumina DNA Library Prep, Illumina Inc.), and paired-end sequencing (2 × 150 bp) was carried out on the Illumina NovaSeq X Plus platform at Novogene (USA). Raw sequence reads were quality-filtered with Trimmomatic v0.39, retaining fragments longer than 50 bp, and quality was assessed using FastQC v0.11.9. High-quality reads were mapped to the FPV reference genome (GenBank accession: NC_002188.1) using BWA-MEM v0.7.17 (22). Alignments were processed with SAMtools v1.12 (23), FreeBayes v1.3.5 (24), and BCFtools v1.12 (25) to generate a high-confidence consensus sequence. Genome annotation was performed using Prokka v1.14.6, and the assembled genome was designated FPV_MGH-AM01.

### Comparative genomic analysis

A total of 23 complete FPV genome sequences were retrieved from the NCBI database. Multiple sequence alignment of these references with FPV_MGH-AM01 was performed using MAFFT v7.1 under default parameters. Pairwise percent identity was calculated from the aligned data set, and a percent identity matrix was generated in Python using Biopython, NumPy, and Pandas. Heatmap visualization was produced with Seaborn and Matplotlib on a Linux platform, highlighting FPV_MGH-AM01. Global percent identity across the aligned genomes was calculated as the proportion of identical nucleotides in non-gap positions. Alignment figures were generated in Python using Biopython and Matplotlib, with FPV_MGH-AM01 displayed as a green reference bar and other sequences shown as blue alignment blocks.

### Phylogenetic analysis

Phylogenetic analysis was performed using two approaches. First, complete genome sequences of 24 FPV strains (including FPV_MGH-AM01) were aligned with MAFFT, and phylogenetic trees were reconstructed in IQ-TREE 2 (v2.1) with 1,000 bootstrap replicates. Second, phylogenetic relationships based on the P4b gene were inferred using 37 reference sequences from GenBank. The best-fit substitution model (GTR + F + I + G4) was applied in IQ-TREE 2 with 1,000 bootstrap replicates. Branches supported by bootstrap values ≥ 70% were considered well resolved and annotated accordingly. Phylogenetic trees were visualized using the iTOL web server (https://itol.embl.de/).

### Mutational analysis and protein structure prediction

The genome sequence of FPV_MGH-AM01 was aligned with the reference sequence (GenBank accession: NC_002188.1) using CLC Sequence Viewer (v8.0). Nucleotide and amino acid substitutions were manually examined. Global Model Quality Estimation (GMQE) scores and tertiary structures of the selected proteins were predicted using the Swiss-Model online server (https://swissmodel.expasy.org/) under default parameters. Predicted protein structures were visualized with PyMOL, and structural validation was performed using PROCHECK via SAVES v6.0 (https://saves.mbi.ucla.edu/) and ProSA-web (https://prosa.services.came.sbg.ac.at/prosa.php), including Ramachandran plot analysis.

### Secondary structure prediction

The secondary structural features of the selected FPV proteins were analyzed using the Self-Optimized Prediction Method with Alignment (SOPMA) tool, available at the Network Protein Sequence Analysis (NPSA) server (https://npsa.lyon.inserm.fr/cgi-bin/npsa_automat.pl?page=/NPSA/npsa_sopma.html`). SOPMA predicts protein conformational states, including α-helices, β-strands, β-turns, and random coils, based on multiple sequence alignments and optimized parameters. Amino acid sequences of the FPV_MGH-AM01 proteins, along with the reference strain (NC_002188.1), were submitted in FASTA format. Predictions were performed using default parameters with a window width of 17, a similarity threshold of 8, and four conformational states considered. The output provided the percentage distribution of amino acids in α-helices, extended strands, β-turns, and random coils.

### Computational antigenicity analysis of FPV proteins

The antigenic potential of selected FPV proteins was assessed using the BepiPred Linear Epitope Prediction tool available at the Immune Epitope Database (IEDB) B-cell epitope prediction server (https://tools.iedb.org/bcell/). Protein sequences of the selected FPV genes-including the serpin gene family protein, V-type Ig domain protein, protein tyrosine phosphatase, and ankyrin repeat protein-were submitted individually to the server. Antigenicity scores were generated for each amino acid residue, with values above the default threshold considered potential B-cell epitopes. Both the newly sequenced FPV_MGH-AM01 isolate and the reference FPV genome (GenBank: NC_002188.1) were analyzed for comparison.

## RESULTS

### Clinical manifestations, molecular detection, and isolation of FPV

**Fig 1:**
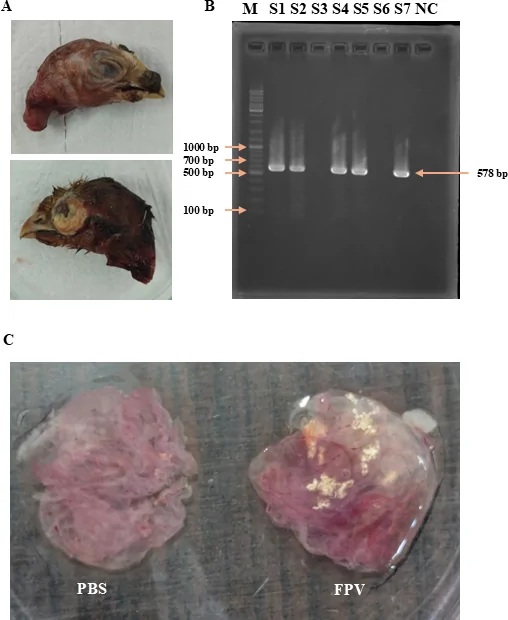
Clinical signs, molecular identification, and FPV isolation. (**A**) Clinical signs. Wart-like nodular proliferative growths on the comb, beak and wattles of affected birds. (**B**) Molecular detection. PCR amplified partial P4b gene of 578 bp run on the 1.5% agarose gel. M means DNA marker; NC means negative control; S means sample. (**C**) Isolation of FPV. The 10-day-old embryonated chickens’ eggs were inoculated through CAM route with the suspected prepared samples and incubated. The thickened CAM showed characteristic pock lesions.

A total of seven samples were collected from layer chicken flocks suspected of FPV infection. The affected birds exhibited wart-like nodules or scabs on the comb, wattles, beak, legs, and other unfeathered regions, along with drooping wings, ocular swelling with discharge, and diphtheritic lesions in the mouth, throat, and upper respiratory tract (Fig. 1A). Among these, five samples tested positive for FPV by PCR using FPV gene-specific primer sets, which produced distinct target bands of 578 bp on agarose gel electrophoresis (Fig. 1B). Virus isolation was attempted from one representative positive sample using embryonated chicken eggs (ECEs). After several blind passages, the fourth passage revealed thickened CAM with characteristic pock lesions, confirming successful viral growth and isolation (Fig. 1C).

### Complete genome characterization and phylogenetic analysis

**Fig 2:**
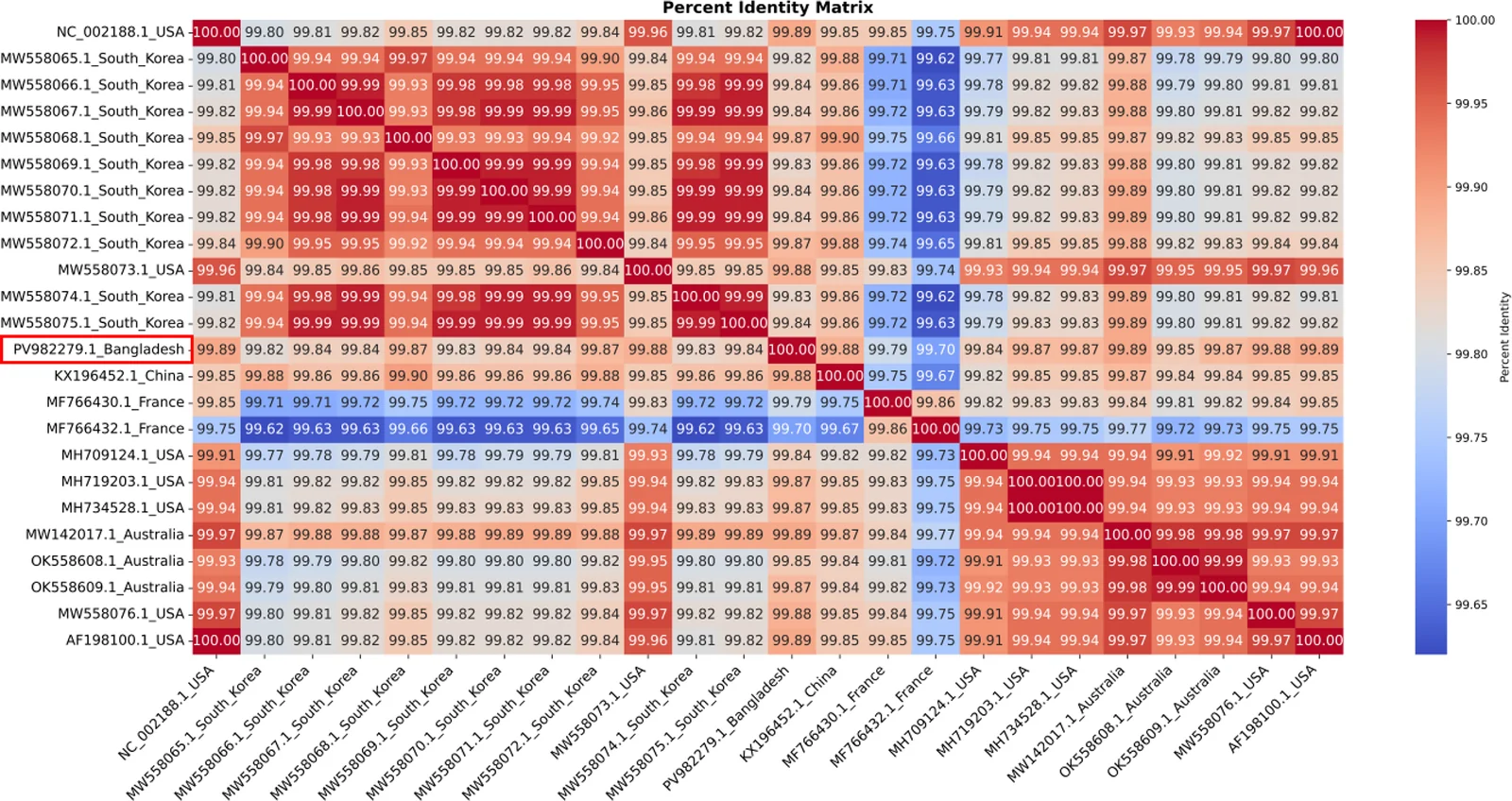
Heat map showing percent identity matrix of the complete nucleotide sequences of 24 FPV strains. A total of 23 complete FPV genome sequences were retrieved from the GenBank. Multiple sequence alignment was performed using MAFFT under default parameters. Percent identity was calculated, and the matrix was constructed in Python using Biopython, NumPy, and Panda’s libraries. The heatmap was visualized under Seaborn and Matplotlib libraries in the Linux operating system. The identified strain of this study was marked by the red box.

**Fig 3:**
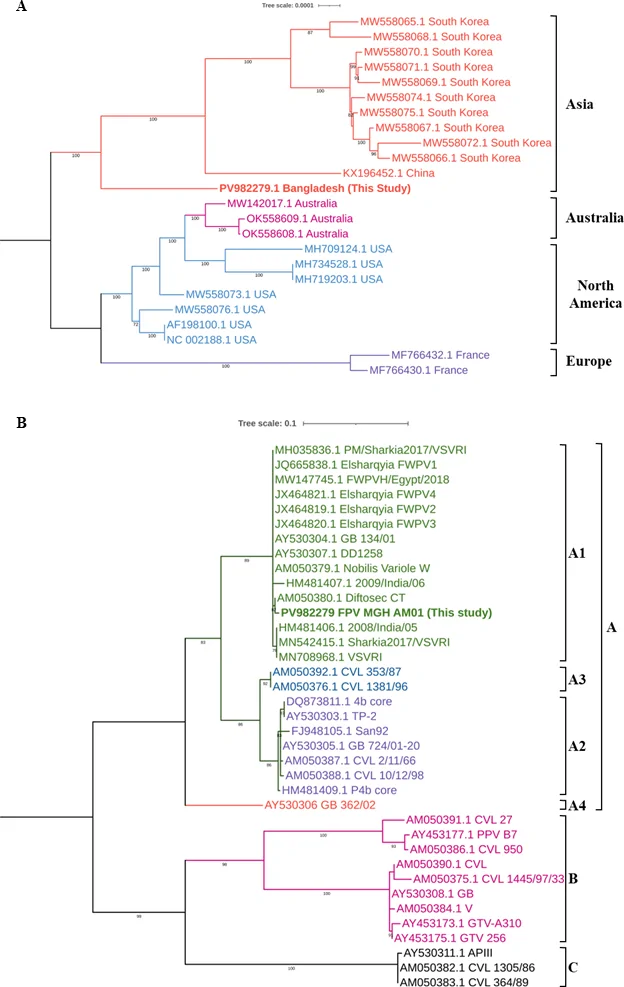
Phylogenetic analysis and clade determination of FPV. Maximum-likelihood phylogenetic trees were constructed using IQ-TREE2 under the GTR + F + I + G4 substitution model with 1,000 bootstrap replicates. Branches supported by bootstrap values ≥ 70% were considered well resolved and are indicated on the trees. The trees were visualized using the iTOL online tool. (**A**) Phylogenetic analysis based on 23 complete FPV genome sequences. The strain identified in this study is highlighted in red to indicate its position relative to reference strains. (**B**) Determination of the FPV clade based on 37 P4b gene sequences. the strain identified in this study is indicated in green bold text.

The complete genome of the isolated FPV was sequenced using next-generation sequencing (NGS). The assembled genome of the isolate, designated FPV_MGH-AM01, was 288,539 bp in length with a GC content of 30.9%. The complete genome encodes 263 ORFs, and 9.24 kbp inverted terminal repeats (ITRs) are present at the 5′ and 3′ terminal regions. BLAST analysis revealed 99.70% nucleotide identity with previously reported FPV strains, including NC_002188.1. The full genome sequence has been deposited in GenBank under accession number PV982279. A percent identity heatmap showed that the Bangladeshi strain (PV982279.1) exhibited pairwise identities ranging from 99.69% (with MF766432.1, France) to 99.89% (with NC_002188.1_USA, MW558073.1_USA, AF198100.1_USA, and MW142017.1_Australia), indicating a high degree of similarity with all reference strains (Fig. 2). Phylogenetic analysis based on complete genome sequences revealed that FPV_MGH-AM01 clustered closely with South Korean strains (Fig. 3A). Lineage analysis further showed that FPV_MGH-AM01 shares regional genetic features with Asian FPV strains but also represents a distinct lineage within the broader global avipoxvirus phylogeny (Fig. 3A). Phylogenetic analysis based on the P4b genes confirmed that the isolate belongs to clade A1 (Fig. 3B). Moreover, the Bangladeshi FPV strain is genetically conserved, retaining genomic regions deleted in certain Chinese and French isolates, and is most closely related to contemporary South Korean strains (Fig. S1).

### Genomic mutations and protein variability

**Fig 4:**
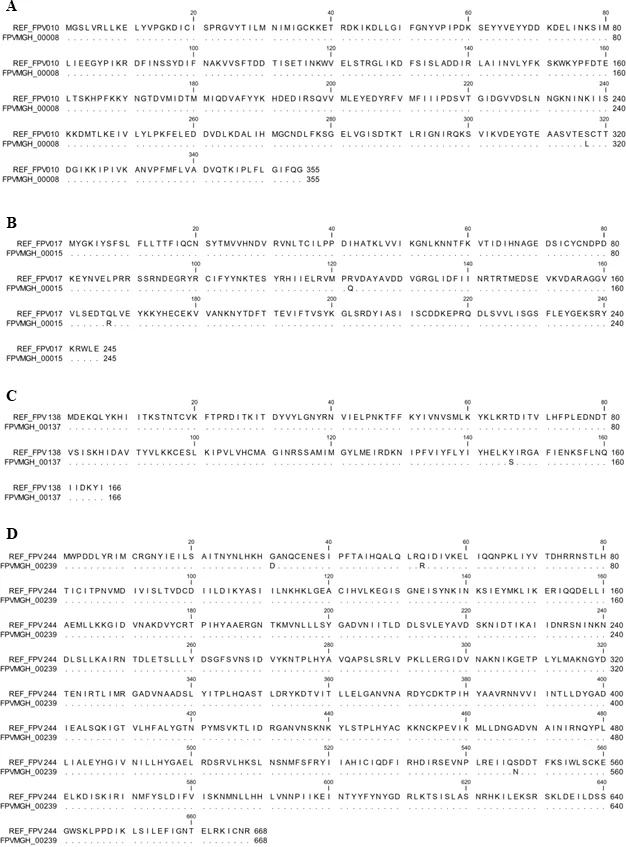
Amino acid substitution analysis of four important viral proteins. (**A**) Serpin gene family protein. (**B**) V-type ig domain. (**C**) Protein tyrosine phosphate. (**D**) Ankyrin repeat protein. The amino acids sequences of the mentioned proteins were aligned with the reference strain (NC_002188.1) using CLC viewer 8.0 software. Dots indicate identical residues.

The complete genome of most FPV strains encodes approximately 260 ORFs, depending on genome length (2). In this study, the genome of FPV_MGH-AM01 was predicted to contain 263 ORFs using Prokka. Comparative mutational analysis with the reference strain (NC_002188.1) revealed that 71 of the 263 genes carried nucleotide-level mutations, of which 54 genes also exhibited amino acid substitutions (Table S1). The GMQE values of the 54 mutated proteins were evaluated to validate the reliability of their predicted structures, enabling accurate structural comparison with the reference and subsequent functional interpretation (Table S2). Among these, around 15 proteins showed notable differences in GMQE scores and sequence identity (Table S2). Based on predicted functional importance, four proteins—serpin, V-type Ig domain protein, protein tyrosine phosphatase, and ankyrin repeat protein—were selected for downstream analyses. In the serpin protein, amino acid substitution S317L was identified in FPV_MGH-AM01 compared with the reference strain (NC_002188.1) (Fig. 4). The V-type Ig domain protein exhibited R122Q and Q167R substitutions, while the protein tyrosine phosphatase showed a Y146S substitution. Additionally, the ankyrin repeat protein family displayed three amino acid changes, G31D, Q53R, and S547N (Fig. 4).

### Physicochemical and structural characterization of important viral proteins

**Fig 5:**
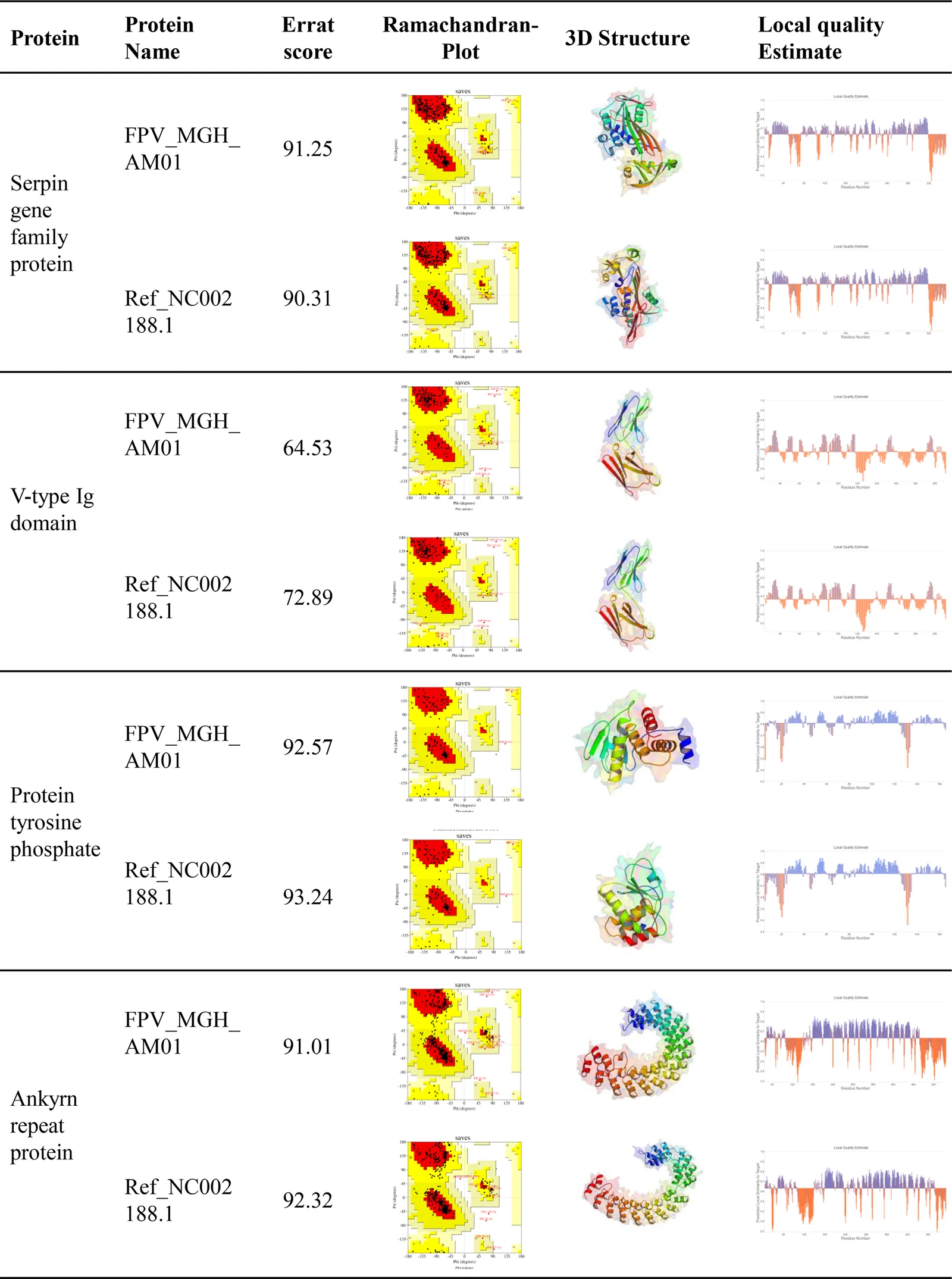
Predicted structural alterations of FPV proteins. Ramachandran plots, 3D structural models, and local quality estimates of the serpin, V-type Ig domain, protein tyrosine phosphatase, and ankyrin repeat proteins of FPV_MGH_AM-01 were generated using Swiss-Model and compared with the reference strain (NC_002188.1).

The physicochemical properties—including molecular weight (MW), theoretical isoelectric point (pI), instability index (II), aliphatic index (AI), and grand average of hydropathicity (GRAVY)—of the serpin, V-type Ig domain, protein tyrosine phosphatase, and ankyrin repeat proteins of FPV_MGH-AM01, as determined by the ProtParam tool, were comparable to those of the reference strain, with only minor strain-specific differences (Table 2). Secondary structure analysis using SOPMA revealed that α-helices and random coils were the predominant structural elements in these proteins of FPV_MGH-AM01 (Table 3). The minimal differences observed between FPV_MGH-AM01 and the reference strain suggest conservation of key structural motifs. Predicted tertiary structures and local quality estimates of the serpin, V-type Ig domain, protein tyrosine phosphatase, and ankyrin repeat proteins were also largely consistent with those of the reference strain (NC_002188.1) (Fig. 5). Ramachandran plot analysis indicated that most residues were positioned in favored regions, with only minor variations likely attributable to the amino acid substitutions identified in the mutational analysis (Fig. 5; Table 4).

**TABLE 2:** Physiochemical properties of serpin gene family protein, V-type Ig domain, protein tyrosine phosphate, and ankyrin repeat protein

Protein	Strain	Instability index (II)	Aliphatic index (AI)	Theoretical pI	GRAVY score	Molecular weight (MW) kDa
Serpin gene family protein	FPV_MGH_AM01(PV982279)	21.70	103.69	5.11	−0.055	40,643.25
	Ref-NC_002188.1	23.16	102.59	5.11	−0.068	40,617.17
V-type Ig domain	FPV_MGH_AM01(PV982279)	42.04	87.80	6.00	−0.368	28,434.32
	Ref-NC_002188.1	40.95	87.80	6.00	−0.368	28,434.32
Protein tyrosine phosphate	FPV_MGH_AM01(PV982279)	30.38	105.60	9.30	−0.028	19,561.12
	Ref-NC_002188.1	30.38	105.60	9.29	−0.031	19,637.22
Ankyrin repeat protein	FPV_MGH_AM01(PV982279)	38.09	112.69	8.08	−0.163	76,314.19
	Ref-NC_002188.1	37.90	112.69	8.08	−0.153	76,201.07

**TABLE 3:** Properties of secondary structure of serpin gene family protein, V-type Ig domain, protein tyrosine phosphate, and ankyrin repeat protein

Protein	Strain	Alpha helix	Extended strand	Random coil
Serpin gene family protein	FPV_MGH_AM01(PV982279)	39.72%	18.03%	42.25%
	Ref-NC_002188.1	41.41%	18.31%	40.28%
V-type Ig domain	FPV_MGH_AM01(PV982279)	9.80%	40.41%	49.80%
	Ref-NC_002188.1	8.98%	40.41%	50.61%
Protein tyrosine phosphate	FPV_MGH_AM01(PV982279)	38.55%	14.46%	46.99%
	Ref-NC_002188.1	40.96%	15.66%	43.37%
Ankyrin repeat protein	FPV_MGH_AM01(PV982279)	44.46%	6.29%	49.25%
	Ref-NC_002188.1	46.11%	6.59%	47.31%

**TABLE 4:** Amino acid residues serpin gene family, V-type Ig domain, protein tyrosine phosphate, and ankyrin repeat protein in Ramachandran plot

Protein	Strain	Most favorable region	Additional allowed region	Generously allowed region	Disallowed region
Serpin gene family protein	FPV_MGH_AM01(PV982279)	89.3%	9.1%	1.6%	0
	Ref-NC_002188.1	90.6%	8.5%	0.9%	0
V-type Ig domain	FPV_MGH_AM01(PV982279)	82.2%	12.6%	2.9%	2.3%
	Ref-NC_002188.1	79.9%	15.5%	4%	4%
Protein tyrosine phosphate	FPV_MGH_AM01(PV982279)	85.4%	13.2%	0.7%	0.7%
	Ref-NC_002188.1	86.1%	12.6%	0.7%	0.7%
Ankyrin repeat protein	FPV_MGH_AM01(PV982279)	83%	15.2%	1.2%	0.6%
	Ref-NC_002188.1	82.9%	15.4%	1.0%	0.6%

### Impact of mutations on protein antigenicity

**Fig 6:**
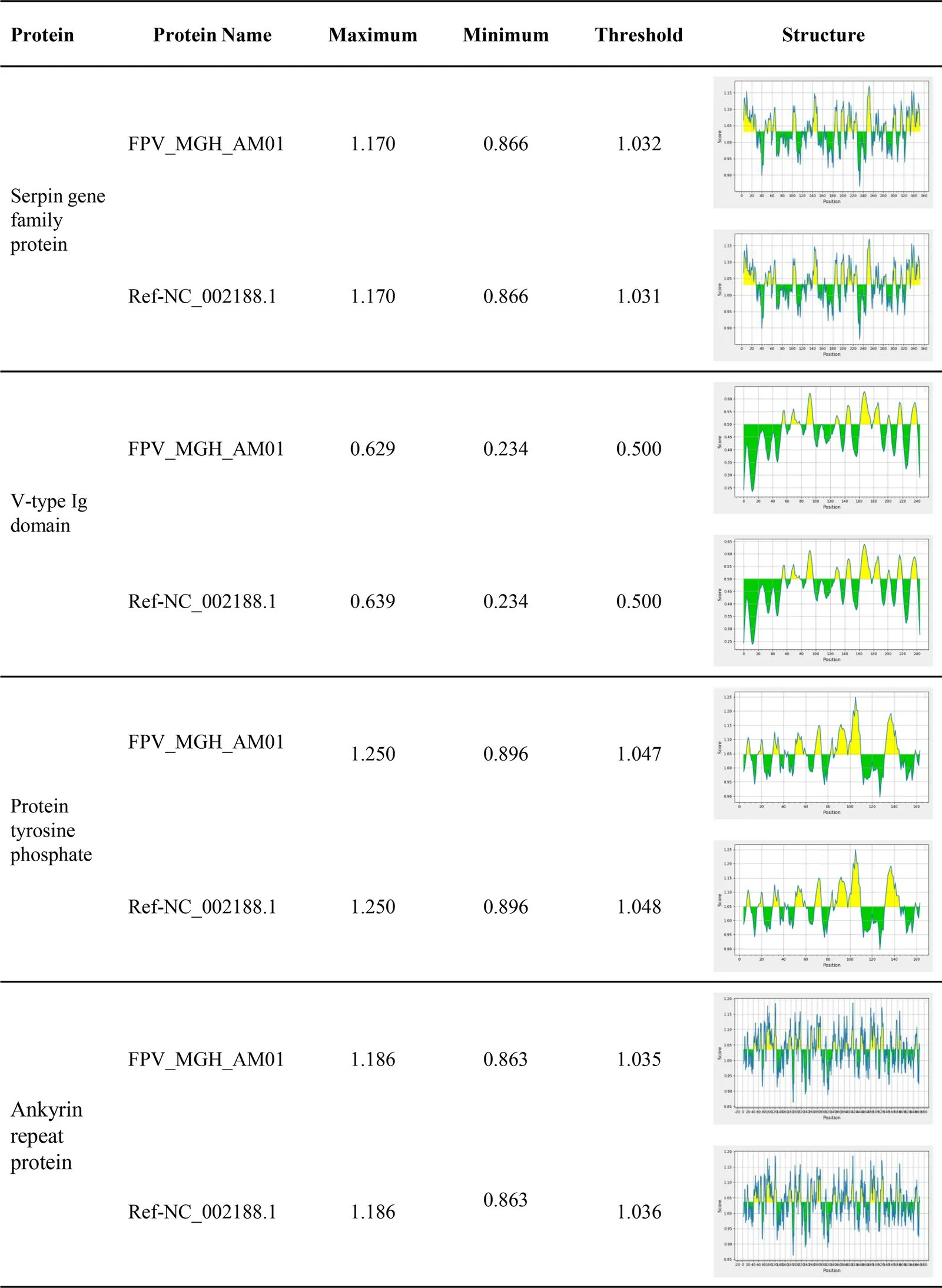
Antigenicity analysis for FPV major proteins. Antigenic properties of the selected proteins were predicted using the Immune Epitope Database (IEDB) analysis resource.

Previous studies have reported that the serpin, V-type Ig domain, protein tyrosine phosphatase, and ankyrin repeat proteins possess antigenic properties (14, 26). Given the mutations identified in these proteins, their antigenicity was further evaluated. The analysis revealed that the V-type Ig domain protein exhibited strong antigenic properties, with altered values compared to the reference strain (Fig. 6). In contrast, the serpin, protein tyrosine phosphatase, and ankyrin repeat proteins of FPV_MGH-AM01 retained antigenic properties but showed only minor changes relative to the reference (Fig. 6).

## DISCUSSION

Fowlpox virus (FPV) continues to pose a significant threat to poultry health worldwide, including Bangladesh, especially in regions with dense poultry populations and suboptimal biosecurity. Despite routine vaccination, outbreaks are still reported, reflecting either incomplete vaccine coverage, antigenic drift, or the presence of genetically divergent field strains (2, 27, 28). Here, we performed the comprehensive molecular identification, virus isolation, and first complete genome sequencing of a FPV from a natural outbreak in Bangladesh, followed by detailed comparative genomic, mutational, and structural analyses of important viral proteins.

FPV infect the host cells by fusion process of viral membranes either with plasma or endosomal membranes and involve the cell signaling pathways over various viral proteins (29). However, the virus then triggers hyperplasia, degeneration, and necrosis that lead to nodular cutaneous growths and fibrinonecrotic lesions on mucosal surfaces (30). Accordingly, the clinical findings of FPV in chickens in the field cases are similar in this investigation. Though clinical symptoms and pathological lesions tentatively diagnosed the FPV infection molecular detection is the gold standard for confirmation of the infection, as viral load in the infected host varies depending on the course of infection (31). This discrepancy of clinical symptoms and viral detection rate can result from several factors, low viral concentration in lesions, during the healing stage, sampling times as well as DNA extraction protocol (32) . In the current investigation, 71.4% (5/7) of samples could be detected by PCR though all the chickens showed clinical symptoms of FPV (18, 32, 33). Development of discrete pock lesions and pronounced thickening of the CAM of the embryonated chickens’ eggs inoculated with FPV is the characteristics of viral growth (4, 34, 35) and accordingly the same virus isolation system was used in this study and found similar lesions in CAM.

Whole-genome sequencing revealed a complete understanding of the genetic makeup, virus evolution, vaccine development of FPV (2). Comparative sequence analysis using a pairwise percent identity matrix is a valuable approach to quantify the genetic similarity between the newly sequenced isolate and previously reported FPV strains (14, 36). This method allows precise assessment of nucleotide-level conservation and divergence across multiple genomes, highlighting both globally conserved regions and strain-specific variations (37) . However, the pairwise identity analysis of the whole genome sequence of our isolate FPV_MGH-AM01 with reference strains from diverse regions, confirming its high genetic stability and placement within a widely distributed lineage (37). Phylogenetic analysis of the fpv167 (P4b) gene classifies avipoxviruses into clades A, B, and C, with clade A subdivided into A1–A3 and lineages, such as Asian, European, American, and Australian (14, 37) . Understanding the clade and lineage of circulating strains is crucial for tracing transmission routes, monitoring viral diversity, and guiding effective vaccine strategies of FPV (38). In our study, the isolate of identified FPV clustered in clade A, Asian lineage, similar to strains reported in neighboring countries like India, Singapore, Iraq and Egypt, suggesting regional circulation and close genetic relatedness (1, 39–41).

Mutational analysis identifies amino acid substitutions that may affect protein structure, function, and host–virus interactions. In FPV_MGH-AM01, 71 genes carried nucleotide changes, with 54 leading to amino acid substitutions. GMQE and sequence identity analyses were used to prioritize proteins with reliable structural predictions for functional interpretation (42) . Such approaches have been applied previously to identify mutations impacting virulence and immune evasion (2). Among the many viral proteins, four—serpin, V-type Ig domain protein, protein tyrosine phosphatase, and ankyrin repeat protein—were selected due to their critical roles in immune modulation and pathogenesis (43, 44). Serpins inhibit host serine proteases, blocking apoptosis and modulating inflammation (45). The S317L substitution in serpin of FPV_MGH-AM01 may affect protease inhibition, similar to functionally significant mutations reported previously (45) . Previous findings showed that several mutations in the V-type Ig domain proteins of FPV identified from India, china and Egypt (18, 46). In the present study, R122Q and Q167R substitutions were detected in the V-type Ig domain proteins; however, their functional impact has not been investigated (46) . The protein tyrosine phosphatases, such as the H1L homolog in vaccinia virus (VV), have been reported to play a crucial role in viral morphogenesis and assembly (2). FPV138 of FPV, a homolog of VV H1L, may employ a similar mechanism for regulating phosphorylation events during virion assembly, which has showed several mutations in this study (2). Ankyrin repeat protein identified in this study showed various amino acid mutations and this protein involved in modulating host immune signaling and influencing viral virulence (2). Additionally, structural analysis of the major FPV proteins by bioinformatics tools indicated minor variations of the metrices, which may beneficial for the further studying viral pathogenesis and vaccine design (47).

Taken together, the first complete genome sequence of FPV isolated from the clinical outbreak of Bangladesh revealed Asian lineage and clade A1 and evolutionarily related with India, Iraq and Egypt. The viral genome was conserved yet harbored distinct nucleotide and amino acid substitutions, particularly in important proteins, such as the serpin, V-type Ig domain, protein tyrosine phosphatase, and ankyrin repeat proteins. Despite these variations, its structural and physicochemical properties remained highly similar to global strains, particularly those from South Korea. The genomic information presented here may support ongoing genomic surveillance of circulating FPV strains and help evaluate the effectiveness of currently used vaccines, as well as aid in the design of improved vaccine candidates. Continued genomic surveillance from field cases at various regions of Bangladesh in comparing with vaccine strains is needed and functional studies on immune-related proteins are essential to guide evidence-based control strategies and mitigate the economic burden of FPV in the Bangladeshi poultry industry.

## SUPPLEMENTAL MATERIAL

Figure S1 spectrum.03114-25-s0001.doc Alignment of complete genomes of 24 FPVs, including the identified strains in this study.

Table S1 spectrum.03114-25-s0002.xlsx Comparative analysis of nucleotide and amino acid mutations in FPV_MGH-AM01 relative to the reference strain (NC_002188.1)

Table S2 spectrum.03114-25-s0003.xlsx Structural model quality estimation (GMQE) and sequence identity of the mutated proteins in the newly identified fowlpox virus genome were analyzed using various tools and compared with the reference strain.

ASM does not own the copyrights to Supplemental Material that may be linked to, or accessed through, an article. The authors have granted ASM a non-exclusive, world-wide license to publish the Supplemental Material files. Please contact the corresponding author directly for reuse.
