# Supplementary material for: First complete genome of a fowlpox virus isolated in Bangladesh reveals Asian lineage and clade A1
Source: Microbiol Spectr. 2026 May 29;14(7):e03114-25. doi: 10.1128/spectrum.03114-25 (PMC13339807; doi:10.1128/spectrum.03114-25)

**Supplementary figure 1. Alignment of complete genomes of 24 FPVs including the identified strains in this study.** Multiple sequence alignment was performed using MAFFT under default parameters. The resulting FASTA alignment file was used to generate an alignment figure. NCBI references were used as query, and each sequence was compared to the query. Global percent identity was calculated as the proportion of identical positions across all non-gap sites, and contiguous aligned regions were displayed as horizontal bars relative to the query. The figure was generated using Python with the Biopython and Matplotlib libraries in the Linux operating system. The query sequence was displayed as a full-length green bar at the bottom, while all other sequences were shown as alignment blocks in blue color.


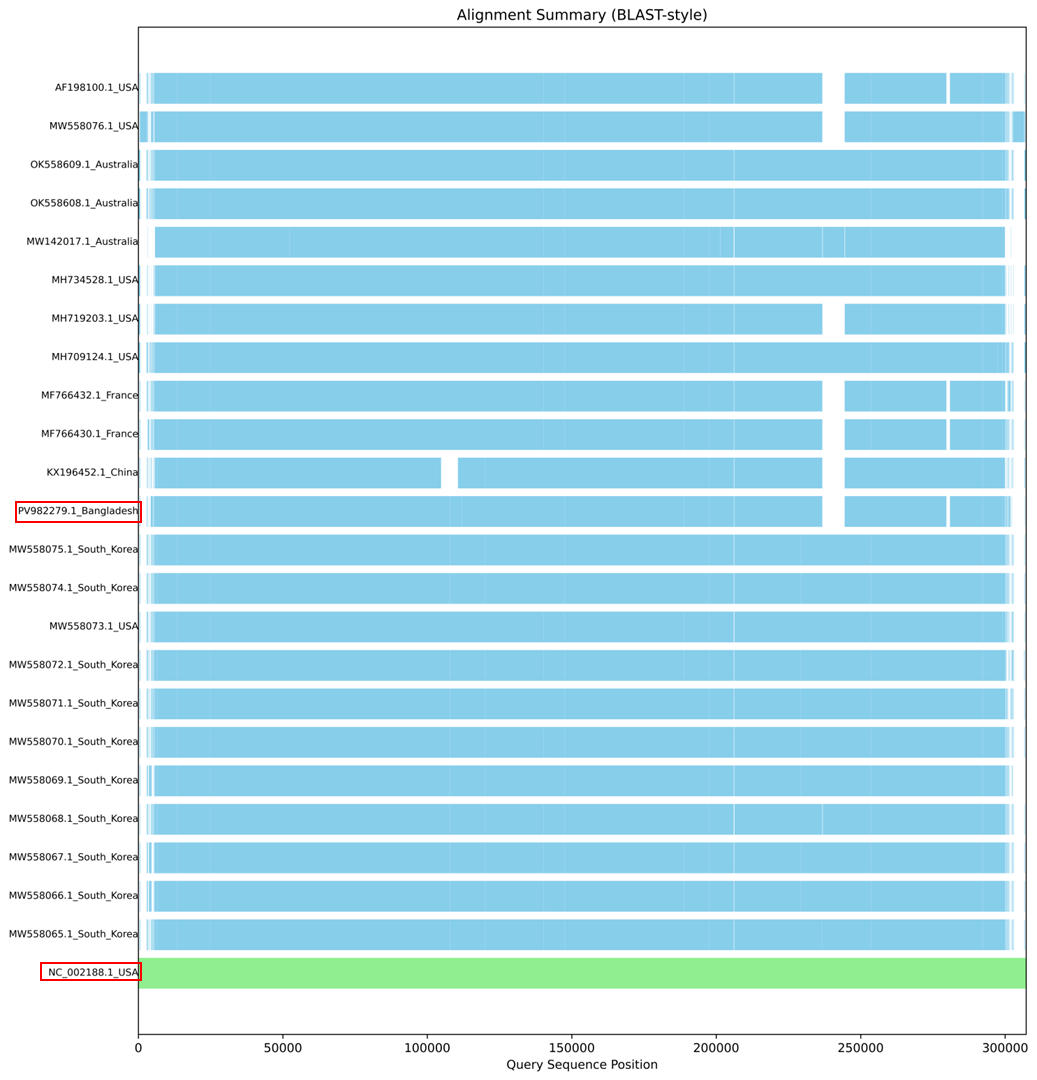

Supplement: Figure S1 — Alignment of complete genomes of 24 FPVs, including the identified strains in this study. [file spectrum.03114-25-s0001.doc]
